# Complement Protein C1q Interacts with DC-SIGN *via* Its Globular Domain and Thus May Interfere with HIV-1 Transmission

**DOI:** 10.3389/fimmu.2016.00600

**Published:** 2016-12-22

**Authors:** Lina Pednekar, Hrishikesh Pandit, Basudev Paudyal, Anuvinder Kaur, Maha Ahmed Al-Mozaini, Lubna Kouser, Berhane Ghebrehiwet, Daniel A. Mitchell, Taruna Madan, Uday Kishore

**Affiliations:** ^1^Biosciences, College of Health and Life Sciences, Brunel University London, Uxbridge, UK; ^2^Department of Innate Immunity, National Institute for Research in Reproductive Health (ICMR), Mumbai, India; ^3^Department of Infection and Immunity, King Faisal Specialist Hospital and Research Centre, Riyadh, Saudi Arabia; ^4^Department of Medicine, State University of New York, Stony Brook, NY, USA; ^5^Clinical Sciences Research Laboratories, University of Warwick, Coventry, UK

**Keywords:** DC-SIGN, C1q, globular head domain, HIV-1, protein–protein interaction

## Abstract

Dendritic cells (DCs) are the most potent antigen-presenting cells capable of priming naïve T-cells. Its C-type lectin receptor, DC-SIGN, regulates a wide range of immune functions. Along with its role in HIV-1 pathogenesis through complement opsonization of the virus, DC-SIGN has recently emerged as an adaptor for complement protein C1q on the surface of immature DCs *via* a trimeric complex involving gC1qR, a receptor for the globular domain of C1q. Here, we have examined the nature of interaction between C1q and DC-SIGN in terms of domain localization, and implications of C1q–DC-SIGN-gC1qR complex formation on HIV-1 transmission. We first expressed and purified recombinant extracellular domains of DC-SIGN and its homologue DC-SIGNR as tetramers comprising of the entire extra cellular domain including the α-helical neck region and monomers comprising of the carbohydrate recognition domain only. Direct binding studies revealed that both DC-SIGN and DC-SIGNR were able to bind independently to the recombinant globular head modules ghA, ghB, and ghC, with ghB being the preferential binder. C1q appeared to interact with DC-SIGN or DC-SIGNR in a manner similar to IgG. Mutational analysis using single amino acid substitutions within the globular head modules showed that Tyr^B175^ and Lys^B136^ were critical for the C1q–DC-SIGN/DC-SIGNR interaction. Competitive studies revealed that gC1qR and ghB shared overlapping binding sites on DC-SIGN, implying that HIV-1 transmission by DCs could be modulated due to the interplay of gC1qR-C1q with DC-SIGN. Since C1q, gC1qR, and DC-SIGN can individually bind HIV-1, we examined how C1q and gC1qR modulated HIV-1–DC-SIGN interaction in an infection assay. Here, we report, for the first time, that C1q suppressed DC-SIGN-mediated transfer of HIV-1 to activated pooled peripheral blood mononuclear cells, although the globular head modules did not. The protective effect of C1q was negated by the addition of gC1qR. In fact, gC1qR enhanced DC-SIGN-mediated HIV-1 transfer, suggesting its role in HIV-1 pathogenesis. Our results highlight the consequences of multiple innate immune pattern recognition molecules forming a complex that can modify their functions in a way, which may be advantageous for the pathogen.

## Introduction

Dendritic cell-specific intracellular adhesion grabbing non-integrin (DC-SIGN) is a C-type lectin expressed on DCs that functions as a pattern recognition receptor (PRR). It can interact with a range of viral, bacterial, and fungal pathogens to primarily promote Th2 responses *via* activation of the mitogen-activated protein kinases Erk1 and Erk2 (1), leading to the clearance of pathogens. DC-SIGN also modulates TLR signalling by activating serine and threonine kinase Raf1, which acetylates the NF-κB subunit p65 upon interaction with pathogens, such as *Mycobacterium tuberculosis, Mycobacterium leprae, Candida albicans*, and measles virus (2, 3). Acetylation of p65 and increased IL-10 transcription leads to an enhanced anti-inflammatory cytokine response (2). DC-SIGN also mediates DC–T cell interaction *via* intracellular adhesion molecule-3 (ICAM-3) (4). In addition, DCs can adhere to endothelial cells expressing high levels of ICAM-2 *via* DC-SIGN. Further interactions between lymphocyte function-associated antigen-1 (LFA-1) and ICAM-1 with ICAM-2–DC-SIGN (5) promote trans-endothelial migration of DCs, allowing them to travel from the blood to the lymphatic system where they can induce T cell responses. Martinez et al. have shown that DC-SIGN stimulated CD3-activated T cells produce IL-2, which, in turn, enhances T cell differentiation (6). DC-SIGN can bind the cell wall component, glycolipid ManLAM of *M. tuberculosis*, and inhibit DC maturation through the suppression of TLR-4 (7). Such a cross talk between TLRs and DC-SIGN that generates anti-inflammatory immune response highlights the two-faced role of DC-SIGN in immune regulation.

DC-SIGN can bind to HIV-1 envelope protein gp120 through glycan structures (8) and mediate HIV transmission in *cis* and *trans* fashion. The *cis* mode supports DC-SIGN-mediated viral internalization and limited replication; in *trans* mode, viral particles are endocytosed and presented to CD4^+^ cells (9). DC-SIGN, thus, allows DCs to carry HIV-1 to the lymph nodes where interactions between DCs and T cells leads to transmission of the virus to CD4^+^ T cells, leading to their infection and eventual depletion (10). The hepatitis C virus (HCV) envelope glycoprotein E2 is another viral protein DC-SIGN engages with (11). This is achieved through utilizing its high quality endocytic capability to internalize the viral antigen, leading to the infection of DCs (12).

Structurally, DC-SIGN is composed of an extracellular domain (ECD), which exists as a tetramer, stabilized by an N-terminal α-helical neck region, followed by a carbohydrate recognition domain (CRD) (8). Its affinity for N-linked high mannose oligosaccharides is evident through its ligands HIV-1 gp120 and ICAM-3 being highly glycosylated, indicating that this binding is mediated through the CRD region (13, 14). Studies have shown that the interaction between gp120 and DC-SIGN triggers a drop in IL-6 production by immature DCs. In addition, gp120 binding to DC-SIGN has also been shown to suppress the anti-apoptotic activity of Nef and induce apoptosis in immature DCs (14). Thus, HIV pathogenesis heavily relies on the interplay of molecular mechanisms involving DC-SIGN.

Recently, it has emerged that DC-SIGN interacts with the complement classical pathway recognition protein, C1q (15), in conjunction with its globular head receptor, gC1qR, on the surface of immature DCs. C1q as well as gC1qR is known to associate with the viral envelope protein gp41 of HIV-1 (16, 17). C1q has been shown to interact with gp41 ectodomain *via* its globular head (gC1q) domain (18), specifically *via* the A chain (19), in a way similar to C1q–IgG interaction (20, 21). Similar to IgG, the ability of gp41 to form aggregates (16) leads to an enhanced activation of the C1 complex, as well as the function of gp41 to initiate the classical pathway on the surface of infected cells in an antibody-independent manner (22). The C1q binding site on gp41 resides within residues 601–613 of the immunodominant loop region (16), which contains hydrophobic side chains and forms a cleft (23).

gC1qR on its own has been shown to suppress the production of HIV-1 in MT-4 and H9 human T cell lines, and macrophages infected with HIV-1_IIIB_ and HIV-1_Ba-L_ (24) Suppression of virus production is further enhanced when gC1qR is preincubated with the target cell lines prior to HIV-1 challenge, suggesting that interference with viral entry by gC1qR occurs through interaction with CD4 (24). gC1qR is known to bind to a range of viral ligands including the HCV core protein (25), adenovirus core protein V (26) EBNA-1 (27), and rubella virus capsid protein (28). gC1qR can also act as a receptor for HIV-1 gp41 and target healthy CD4^+^ T cells to natural killer (NK) cell-mediated lysis (17). This bystander effect of autologous killing occurs through the surface translocation of NKP44L, *via* activation of PI3K, NADPH oxidase, and p190 RhoGAP.

Recently, DC-SIGN, C1q, and gC1qR on immature DCs have been shown to form a tripartite complex, with a plausible role in DC differentiation through signaling *via* the NF-κB pathway (15). Given that each of these innate immune proteins (C1q, DC-SIGN, and gC1qR) can bind HIV-1, we set out to dissect the nature of interaction between C1q and DC-SIGN and examine how it can impact upon HIV-1 transmission.

## Materials and Methods

### Expression and Purification of Soluble DC-SIGN and DC-SIGNR

The pT5T constructs expressing tetrameric and monomeric forms of DC-SIGN and DC-SIGNR were transformed into *Escherichia coli* BL21 (λDE3) (8). Protein expression was performed using bacterial culture in Luria-Bertani medium containing 50 µg/mL of ampicillin at 37°C until OD_600_ reached 0.7. The bacterial culture was induced with 10 mM isopropyl-β-d-thiogalactoside (IPTG) and incubated for a further 3 h. Bacterial cells (1 L) were centrifuged at 4,500 × *g* for 15 min at 4°C and cell pellet was treated with 22 mL of lysis buffer containing 100 mM Tris, pH 7.5, 0.5 M NaCl, lysozyme (50 µg/mL), 2.5 mM EDTA, pH 8.0, and 0.5 mM phenylmethylsulfonyl fluoride (PMSF), and left to stir for 1 h at 4°C. Cells were then sonicated for 10 cycles for 30 s with 2 min intervals and the sonicated suspension was spun at 10,000 *g* for 15 min at 4°C. The inclusion bodies, present in the pellet, were solubilized in 20 mL of 6 M urea, 10 mM Tris–HCl, pH 7.0, and 0.01% β-mercaptoethanol by rotating on a shaker for 1 h at 4°C. The mixture was then centrifuged at 13,000 × *g* for 30 min at 4°C, and the supernatant was drop-wise diluted fivefold with loading buffer containing 25 mM Tris–HCl pH 7.8, 1 M NaCl, and 2.5 mM CaCl_2_ with gentle stirring. This was then dialyzed against 2 L of loading buffer with three buffer changes every 3 h. Following further centrifugation at 13,000 × *g* for 15 min at 4°C, the supernatant was loaded onto a mannan-agarose column (5 mL; Sigma) pre-equilibrated with the loading buffer. The column was washed with five bed volumes of the loading buffer, and the bound protein was eluted in 1 mL fractions using the elution buffer containing 25 mM Tris–HCl pH 7.8, 1 M NaCl, and 2.5 mM EDTA. The absorbance was read at 280 nm, and the peak fractions were frozen at −20°C. Purity of protein was analyzed by 15% w/v SDS-PAGE.

### Expression and Purification of Recombinant Wild-Type Globular Head Modules ghA, ghB, and ghC of Human C1q and Their Substitution Mutants

The recombinant globular head regions of human C1q, ghA, ghB, and ghC modules (19) and their respective mutants (29) were expressed in *E. coli* BL21 as fusion to maltose-binding protein (MBP). Bacterial cells were grown in 200 mL LB medium containing ampicillin (100 µg/mL) at 37°C, and induced with 0.4 mM IPTG at OD_600_ of 0.6 for 3 h, and then centrifuged (4,500 × *g* for 15 min). The cell pellet was suspended in 25 mL of lysis buffer (20 mM Tris–HCl pH 8.0, 0.5 M NaCl, 1 mM EDTA, 0.2% v/v Tween 20, 5% glycerol, 0.1 mM PMSF, and 0.1 mg lysozyme) and incubated at 4°C for 1 h on a rotary shaker. Cell suspension was then sonicated for 30 s with 2 min gaps for 10 cycles. After centrifugation (13,000 × *g* for 15 min), the supernatant was diluted fivefold in buffer I (20 mM Tris–HCl, pH 8.0, 100 mM NaCl, 0.2% Tween 20, 1 mM EDTA, and 5% glycerol), passed through an amylose resin column (15 mL; New England Biolabs), and then washed with three bed volumes of buffer I followed by buffer II (50 mL of buffer I without Tween 20). The protein was then eluted in 1 mL fractions with 10 mM maltose in 100 mL of buffer II and frozen at −20°C after determining protein concentration and purity *via* Nanodrop and 10% w/v SDS-PAGE, respectively.

### Purification of Human C1q from Plasma

C1q was purified from freshly thawed plasma as described previously (19). Briefly, plasma was made 5 mM EDTA, pH 7.5, and centrifuged to remove aggregated lipids. It was then incubated with non-immune IgG coupled to CNBr-activated Sepharose (GE Healthcare, UK) for 1 h at 4°C. The plasma with IgG-Sepharose was filtered through a sintered glass funnel, and C1q-bound Sepharose was then washed extensively with 10 mM HEPES, 140 mM NaCl, 0.5 mM EDTA, pH 7.0. C1q was eluted with CAPS (N-cyclohexyl-3-aminopropanesulfonic acid) buffer (100 mM CAPS, 1 M NaCl, 0.5 mM EDTA, pH 11). The eluted C1q was then passed through a HiTrap protein G column (PierceNet, USA) to remove IgG contaminants and dialyzed against the washing buffer.

### Expression and Purification of Human gC1qR

The recombinant mature gC1qR protein containing 74–282 residues was expressed in *E. coli* BL21 (λDE3) (30). Bacterial cells were grown in 250 mL of LB at 37°C until an OD_600_ of 0.6 was reached and induced with 0.5 mM IPTG. After 3 h, the bacterial cell culture was spun down (4,500 *g* for 15 min). The cell pellet was treated with lysis buffer (20 mM Tris–HCl pH 8.0, 0.5 M NaCl, 1 mM EDTA, 0.2% Tween, 5% glycerol, 0.1 mg lysozyme) and incubated for 1 h at 4°C with shaking. The cell lysate was then sonicated for 10 cycles at 30 s with 2 min intervals. The lysate was spun down at 13,000 *g* for 15 min, and the supernatant was collected and dialyzed for 2 h against 20 mM Tris–HCl, pH 7.5. The dialyzed protein was subjected to ion exchange using a DEAE column and gC1qR eluted at a peak of 0.45 M NaCl.

### Direct Binding ELISA

Microtiter wells were coated overnight at 4°C with DC-SIGN or DC-SIGNR (5, 2.5, 1.25, and 0.625 µg/well) in carbonate–bicarbonate buffer, pH 9.6, and left overnight at 4°C. Wells were blocked with 100 µL of 2% w/v BSA in PBS for 2 h at 37°C. Following three washes with PBS + 0.05% Tween 20, ghA, ghB, ghC, or its substitution mutants (2.5 µg/100 μl) was added to each well in the buffer containing 50 mM NaCl, 100 mM Tris–HCl, pH 7.5, and 5 mM CaCl_2_. MBP (Sigma) was used as a negative control. The plate was incubated at 37°C for 1.5 h and then at 4°C for 1.5 h. The wells were washed and the bound protein was detected with anti-MBP monoclonal antibodies in PBS (1:5,000, Sigma) followed by rabbit anti-mouse IgG-HRP conjugate (1:5,000; Sigma) for 1 h. The color was developed using o-phenylenediamine dihydrochloride (OPD, Sigma) and read at 415 nm.

### Competitive ELISA

DC-SIGN and DC-SIGNR proteins were coated on microtiter wells by overnight incubation at 4°C using 5 µg/well (in 100 µL) in carbonate–bicarbonate buffer pH 9.6. Wells were blocked with 2% BSA in PBS for 2 h at 37°C. Following washing with PBS + 0.05% Tween, the plate was incubated with a steady concentration (5 µg/well) of one competing protein (gC1qR) and various concentrations (5, 2.5, 1.25, 0.625 µg/well) of the second competing protein (ghB) in calcium buffer to give a total of 100 µL/well. After incubating for 1.5 h at 37°C and 1.5 h at 4°C, the wells were washed and anti-gC1qR polyclonal antibody (1:1,000) in PBS was added and incubated for a further 1 h at 37°C. Bound protein was detected by Protein A-HRP conjugate (1:5,000), and the color was developed using OPD. Data were plotted to determine inhibition values of competitive ligand binding.

In order to examine if C1q and globular head modules can inhibit binding of DC-SIGN to gp120, microtiter wells were coated with 250 ng of gp120 (Abcam) in carbonate–bicarbonate buffer and left overnight at 4°C. Plate was blocked with 2% w/v BSA in PBS for 2 h at 37°C, followed by washing three times with PBS + 0.05% Tween 20. Various concentration of ghA, ghB, ghC, and C1q (10, 5, 2.5, 0 µg/mL) were co-mixed with 2.5 µg/mL of DC-SIGN and added to wells in calcium buffer (100 µL/well). After incubation for 1 h at 37°C and 1 h at 4°C, the wells were washed again three times using PBS + 0.05% Tween 20. The binding of DC-SIGN to gp120 in the presence of globular heads and C1q was detected using rabbit anti-DC-SIGN antibody (1:500) and probed with Protein A-HRP conjugate (1:5,000). The color was developed using 3,3′,5,5′-tetramethylbenzidine (TMB) and read at 450 nm spectrophotometrically.

### Western Blotting

Recombinant ghA, ghB, and ghC modules (15 µg), in addition to MBP and BSA as negative control proteins, were run separately on a 12% SDS-PAGE gel and transferred onto PDVF membrane for 1 h at 320 mA. Membrane was blocked in 5% non-fat milk (1 h at room temperature) and 50 µg of recombinant DC-SIGN (tetramer) in loading buffer (25 mM Tris–HCl, pH 7.8, 1 M NaCl, 2.5 mM CaCl_2_) was added and incubated overnight at room temperature. The blot was washed three times for 10 min each in PBS containing 0.05% Tween 20 and then incubated with anti-DC-SIGN (1:1,000) polyclonal antibody (ProSci) in 1% non-fat milk (2 h at 37°C). Following subsequent washes, the membrane was incubated with Protein A-conjugated HRP (1:1,000) (1 h at room temperature). The blot was developed using 3, 3′-diaminobenzidine (DAB; Sigma D7679) as a substrate.

### Fluorescent Microscopy

#### Binding of C1q Globular Head Modules to DC-SIGN Expressed on HEK Cells

DC-SIGN-expressing HEK 293 (DC-HEK) cells, as reported by Lang et al. (31) were grown in DMEM-F12 (Life Technologies, UK) containing 10% v/v FCS and blasticidin (5 µg/mL) (Gibco). The cells were grown on 13 mm glass cover slips till a monolayer of cells was formed and then incubated with 15 µg/mL of recombinant ghA, ghB, and ghC (MBP as a negative control) separately in serum free medium and left to incubate for 30 min in 37°C. Cells were washed with PBS and fixed using 4% v/v paraformaldehyde for 10 min, rinsed again with PBS three times, and then blocked with 5% FCS for 30 min. The slides were incubated for 30 min with mouse anti-MBP antibody to detect MBP fusion proteins and rabbit anti-DC-SIGN antibody to reveal expression of DC-SIGN in DC-HEK cells. After three washes for 30 min each and incubation with secondary antibodies: Alexa Fluor 568 conjugated goat anti-mouse antibody (Thermo Fisher) and Alexa Fluor 488 conjugated goat anti-rabbit antibody (Abcam) for 30 min, the slides were then washed in PBS, mounted, and observed under Leica DM4000 Fluorescent microscope using Leica Application Suite.

#### HIV-1 Transfer Assay with DC-HEK Cells and Pooled Peripheral Blood Mononuclear Cell (PBMC)

Pooled peripheral blood mononuclear cells (HiMedia Laboratories, India) were cultured in RPMI 1640 medium (Sigma Aldrich) containing 10% FBS, 1% Penicillin–Streptomycin (Complete RPMI medium), and stimulated with 5 µg/mL phytohemaglutinin (PHA) and 10 U/Ml of recombinant-human IL-2 (Gibco) for 24 h. PHA/IL-2 was washed off and activated PBMCs were cultured further for 3 days in complete RPMI 1640 medium.

DC-HEK cells were grown and maintained in DMEM-F12 (Sigma-Aldrich, USA) containing 10% FBS and blasticidin (5 µg/mL) (Gibco). Cells were sub-cultured every 3 days and those in the log phase were used for assays. DC-HEK cells were grown in a 12 well plate until 80% confluence. Indicated concentrations of C1q, ghA, ghB, ghC, and gC1qR individually, or in combination, in medium containing 5 mM CaCl_2_ were added to each well and incubated for 2 h to allow binding. Excess protein was removed and cells were challenged with 5 ng/mL p24 of HIV-1 SF-162 strain (kindly provided by Dr. Jay Levy, NIH AIDS Program, National Institutes of Health, USA) for 1 h. MBP was added along with the virus as a negative protein control. Unbound virus was washed off and cells were co-cultured with PHA/IL-2 activated PBMCs for 24 h to facilitate viral transfer. PBMCs in the supernatant were then separated from the adhered DC-HEK monolayer and cultured further for 7 days and viral titer was determined using HIV-1 p24 antigen ELISA of supernatants collected on days 4 and 7 (XpressBio Life Science Products, Frederick, MD, USA). That the reduction in p24 levels was not due to cellular death was confirmed by MTT assay of cultured PBMCs on day 7.

### Statistical Analysis

Viral transfer experiment data were plotted using GraphPad Prism version 5.0, and analyzed for statistical significance using one-way ANOVA. *P* < 0.05 was considered as statistically significant.

## Results

### Both DC-SIGN and DC-SIGNR Bind to C1q

**Figure 1:**
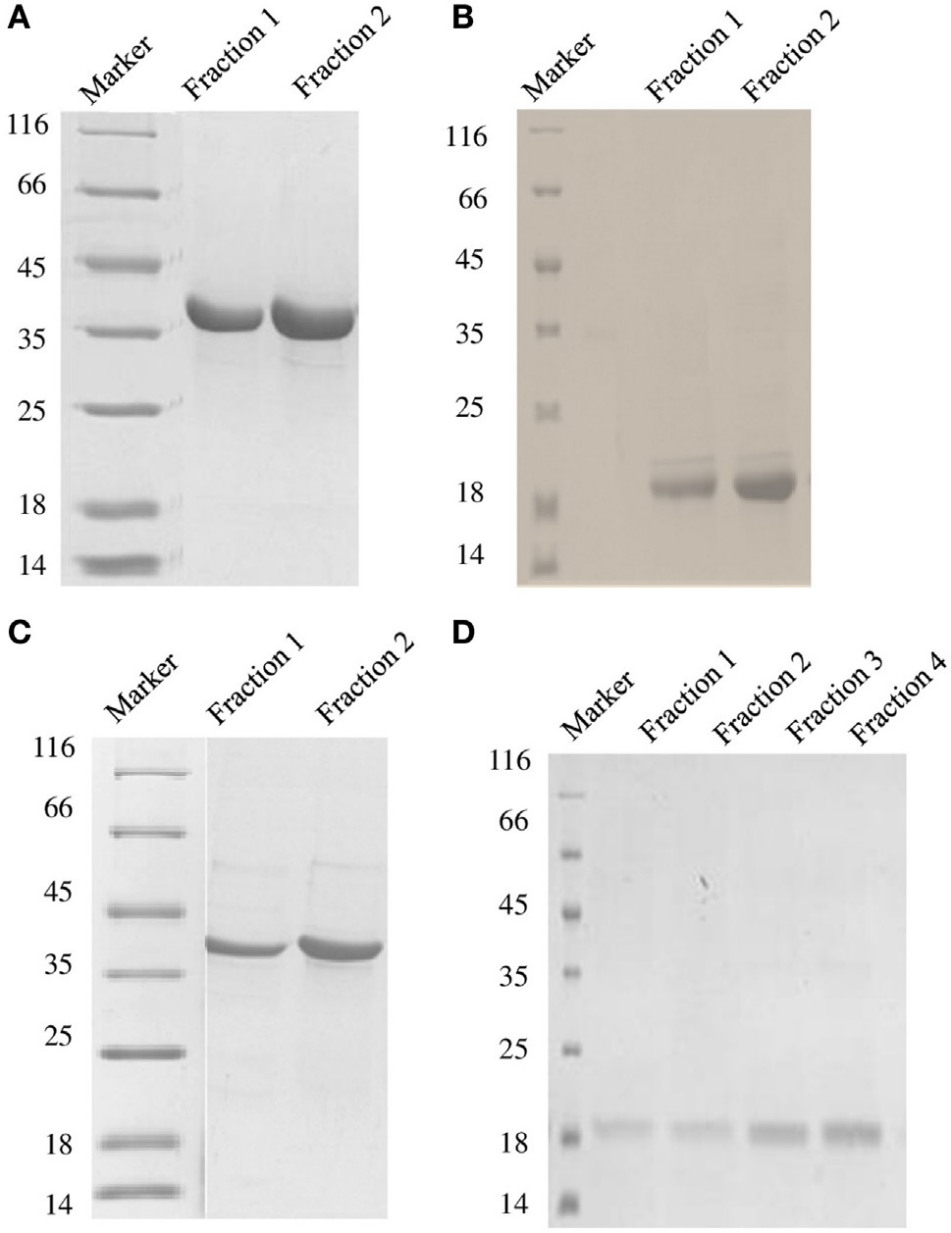
**SDS-PAGE under reducing conditions (12% v/v) showing purified fractions of soluble DC-SIGN tetramer (A), DC-SIGN monomer (B), DC-SIGNR tetramer (C), and DC-SIGNR monomer (D), following purification by mannose-agarose affinity chromatography**.

**Figure 2:**
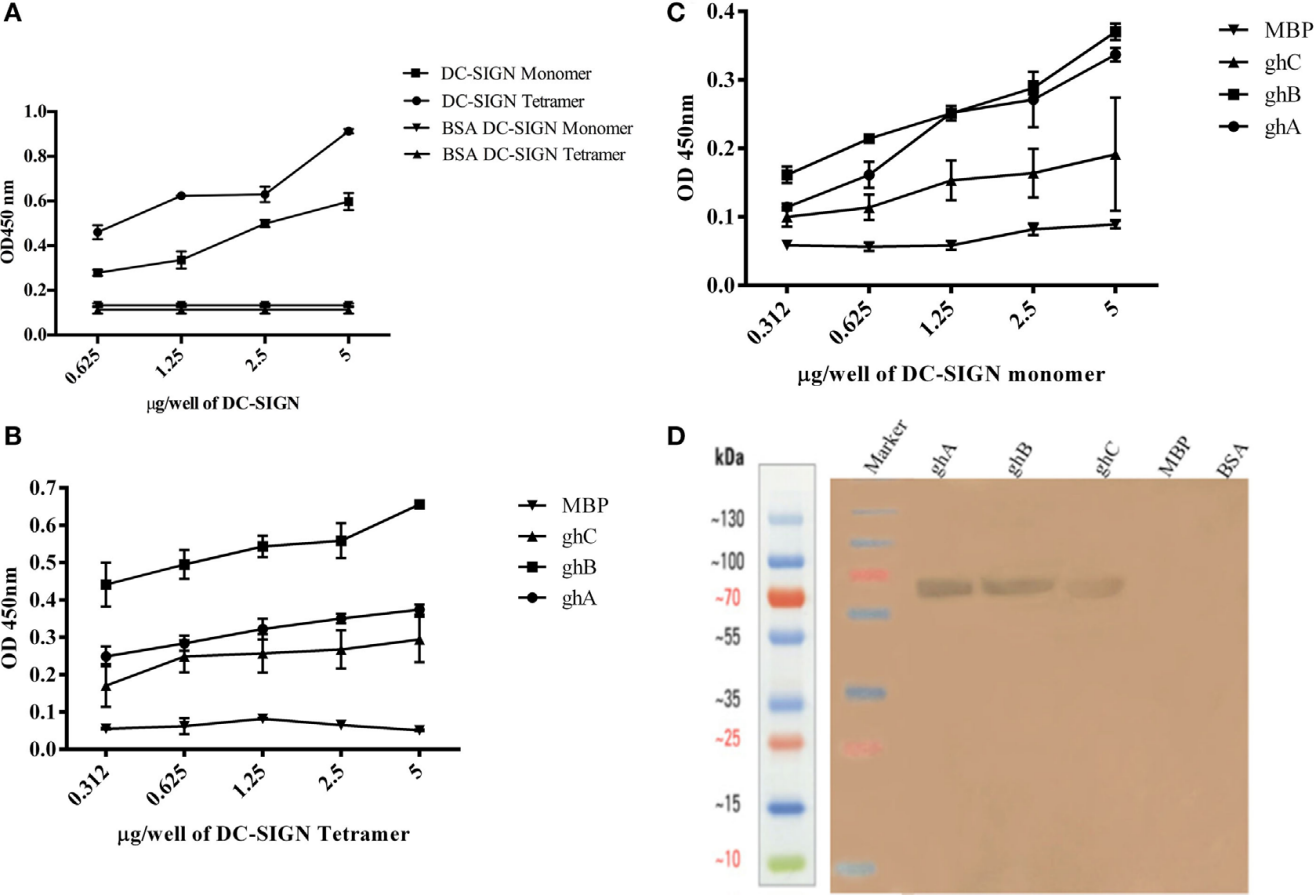
**Interaction of C1q, ghA, ghB, and ghC with DC-SIGN tetramer and monomer**. **(A)** Microtiter wells coated with different concentrations (5, 2.5, 1.25, 0.625 µg/well) of DC-SIGN tetramer or monomer were probed with 2 µg/well of C1q. Bound C1q was detected with anti-C1q polyclonal antibodies (1:1,000 in PBS) and Protein A HRP conjugate (1:1,000 in PBS). BSA was used as a negative control protein. **(B)** Binding of ghA, ghB, and ghC to DC-SIGN tetramer and **(C)** DC-SIGN monomer involved coating a range of concentrations of the respective proteins on microtiter wells, which were then incubated with a fixed concentration of ghA, ghB, ghC, and MBP (2.5 µg/well in 5 mM CaCl_2_ buffer) at 37°C. Binding was detected using anti-MBP monoclonal antibodies (1:5,000 in PBS) and then rabbit anti-mouse IgG-HRP (1:5,000 in PBS). **(D)** Far western blot to show DC-SIGN tetramer binding to membrane-bound ghA, ghB, and ghC: 15 µg of ghA, ghB, and ghC (BSA and MBP as negative control proteins) were run on a 12% SDS-PAGE gel and then transferred on to nitrocellulose membrane. The blot was incubated with 50 µg of DC-SIGN in PBS overnight at room temperature. The bound DC-SIGN protein was detected using anti-DC-SIGN polyclonal antibodies and Protein A HRP conjugate. Bands were developed using diaminobenzidine tablets dissolved in water.

**Figure 3:**
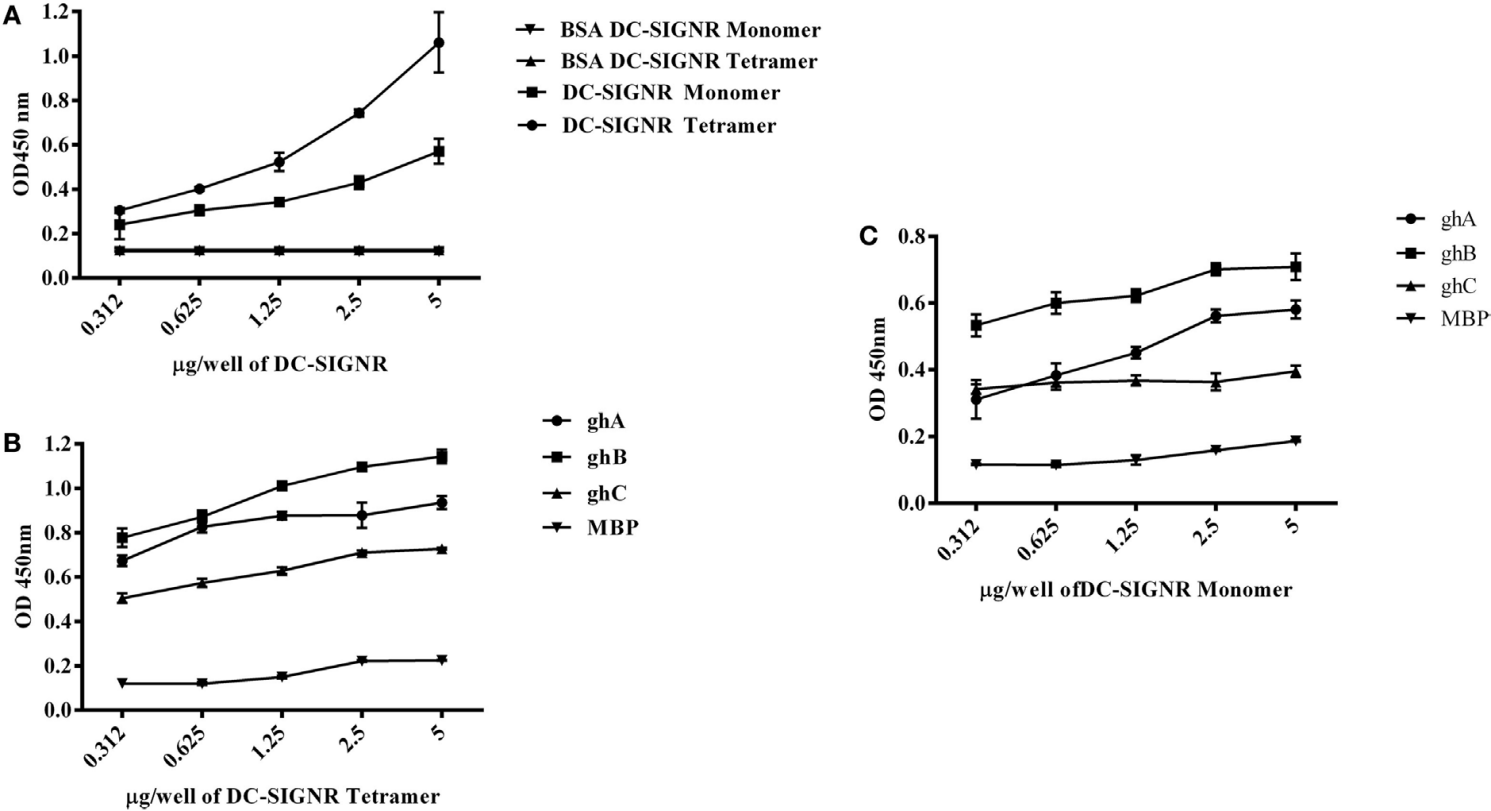
**Interaction of C1q, ghA, ghB, and ghC with DC-SIGNR tetramer and monomer**. **(A)** ELISA to examine binding of C1q to DC-SIGNR tetramer and SIGN-R monomer: DC-SIGNR tetramer or monomer were coated at different concentrations, followed by addition of 2 µg/well of C1q. Bound C1q was probed with anti-C1q polyclonal antibodies (1:1,000 in PBS) and Protein A HRP (1:1,000 in PBS), and the color was developed using o-phenylenediamine dihydrochloride. **(B)** Binding of ghA, ghB, and ghC to DC-SIGNR tetramer and **(C)** SIGN-R monomer: different concentrations of DC-SIGNR tetramer **(B)** and DC-SIGNR monomer **(C)** were coated on microtiter wells in carbonate buffer and incubated overnight at 4°C and then incubated with ghA, ghB, ghC, and MBP (2.5 µg/well in 5 mM CaCl_2_ buffer). Binding was detected using anti-MBP monoclonal antibody and rabbit anti-mouse IgG-HRP conjugate.

DC-SIGN and DC-SIGNR comprising of the entire ECD (Figures 1A,C) and the CRD region alone (Figures 1B,D) were expressed in *E. coli* and affinity-purified on mannose-agarose. The CRD regions of DC-SIGN and DC-SIGNR bound mannose weakly as majority of the proteins appeared in the flow through. The ECD domains of both DC-SIGN and DC-SIGNR bound to mannose with much greater affinity in the presence of Ca^2+^ and eluted with EDTA. Previously, Kang et al. have shown that DC-SIGNR interacts with C1q (32). Recently, work by Hosszu et al. revealed that DC-SIGN bound directly to C1q (15). Thus, we examined direct binding of both the tetrameric and monomeric variants of DC-SIGN and DC-SIGNR with purified human C1q on microtiter plates. Both DC-SIGN (Figure 2A) and DC-SIGNR (Figure 3A) in their tetrameric and monomeric forms were able to bind to C1q in a dose-dependent manner. Experiment showed a strong binding of the tetramers to C1q when compared to the CRD region alone, with the ability of C1q to bind nearly 50% more when the α-helical neck was intact. C1q bound to DC-SIGNR (Figure 3A) better than DC-SIGN (Figure 2A). The ability of the globular head modules to bind DC-SIGN was also examined *via* a far-western blot (Figure 2D), where ghA, ghB, and ghC, run on a SDS-PAGE and transferred on a nitrocellulose membrane, were probed with soluble DC-SIGN tetramer. ghA and ghB appeared to bind DC-SIGN well compared to the ghC module. MBP and BSA, used as negative control proteins, did not bind DC-SIGN tetramer.

### DC-SIGN and DC-SIGNR Neck Region Is Required for Efficient Binding to C1q and Individual Globular Head Modules

Tetrameric (Figure 2B) and monomeric (Figure 2C) DC-SIGN and DC-SIGNR (Figures 3B,C) coated on microtiter wells were probed with ghA, ghB, and ghC to examine whether these globular head modules were able to bind to the ECD and CRD with similar avidity. ghA, ghB, and ghC bound with much greater affinity to DC-SIGN and DC-SIGNR tetramer in comparison to the monomers, indicating that the neck is required for the individual globular heads to bind efficiently. Like C1q, the ghA, ghB, and ghC modules bound DC-SIGNR better than DC-SIGN (Figure 3B).

### DC-SIGN and DC-SIGNR Bind Preferentially to ghB Module

Since C1q bound to DC-SIGN and DC-SIGNR *via* its globular head region, as evident from the use of ghA, ghB and ghC modules, we sought to map their specificity for DC-SIGN and DC-SIGNR binding (Figures 2B and 3B). When DC-SIGN and DC-SIGNR were coated on microtiter wells and probed with ghA, ghB, and ghC, all three globular heads bounds DC-SIGN and DC-SIGNR in a dose-dependent manner, indicating that all three heads are capable of binding to the ligands independently. Furthermore, ghB module was preferential in binding to DC-SIGN (Figure 2B); it bound much better to DC-SIGN compared to ghA and ghC. In addition, ghB was a better binder of DC-SIGNR, as was ghA for DC-SIGNR (Figure 3B) than DC-SIGN (Figure 2B), compared with the ghC module. Although binding of ghA, ghB, and ghC to the CRD domain was significantly lower, the ghB module was still a better binder of DC-SIGN (Figure 2C) and DC-SIGNR (Figure 3C) monomers.

### The ghA Substitution Mutants Bind Differentially to DC-SIGN and DC-SIGNR

**Figure 4:**
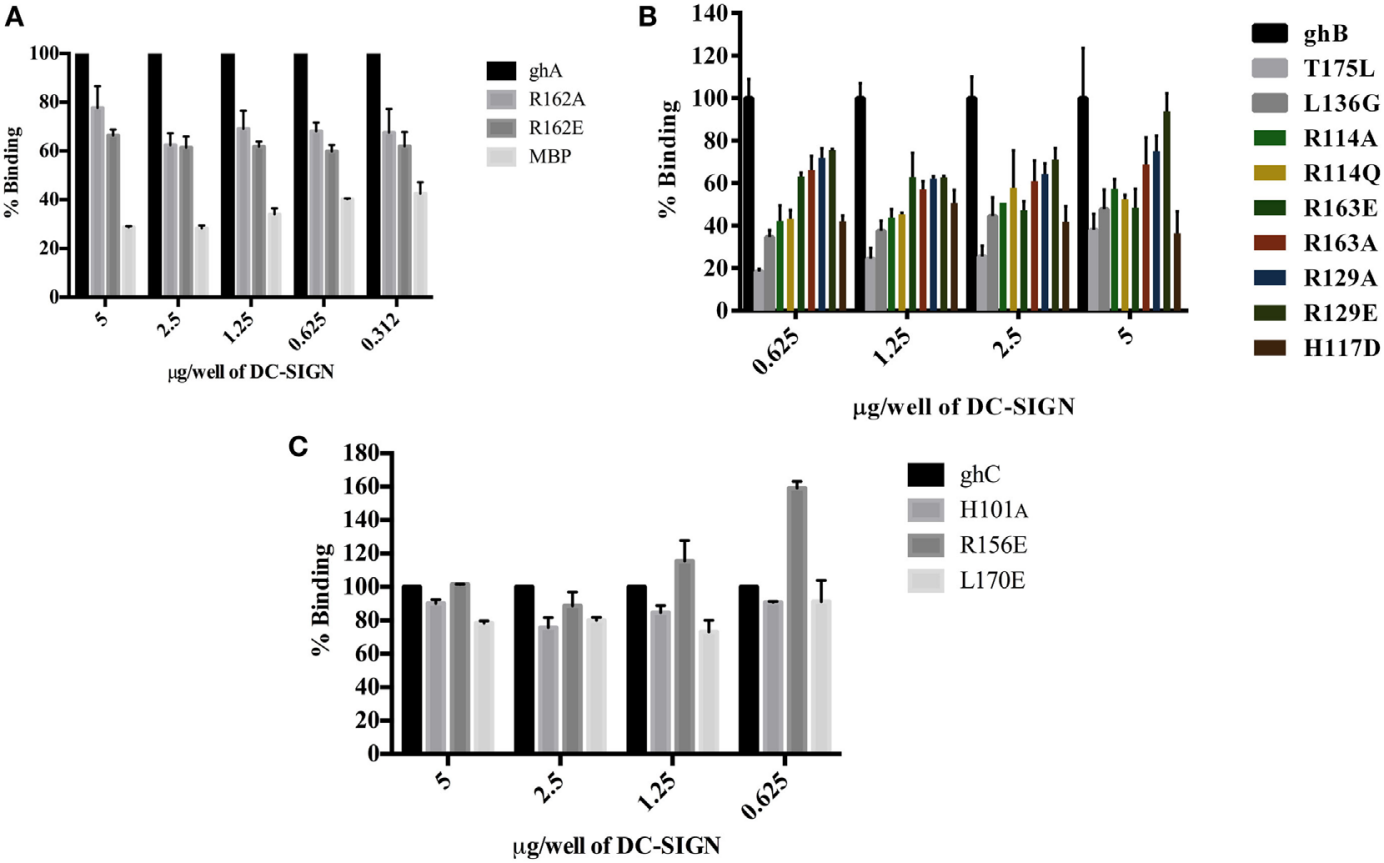
**Binding of globular head substitution mutants to DC-SIGN**. Different concentrations of DC-SIGN tetramer were coated on microtiter wells in carbonate buffer overnight at 4°C. Wells were then incubated with 2.5 µg/well (in CaCl_2_) of recombinant globular head wild type and mutant proteins and probed with anti-MBP monoclonal antibody and rabbit anti-mouse IgG-HRP conjugate. Percent binding was calculated for each mutant using binding of the wild-type globular head module as 100%. **(A)** Binding of ghA, ghA-R162E, and ghA-R162A to DC-SIGN; **(B)** binding of ghB mutants ghB-L136G, ghB-T175L, ghB-R114Q, ghB-R114A, ghB-R163A, ghB-R163E, ghB-R129E, ghB-R129A, and ghB-H117D to DC-SIGN; **(C)** binding of ghC mutants ghC-R156E, ghC-L170E, and ghC-H101A to DC-SIGN.

**Figure 5:**
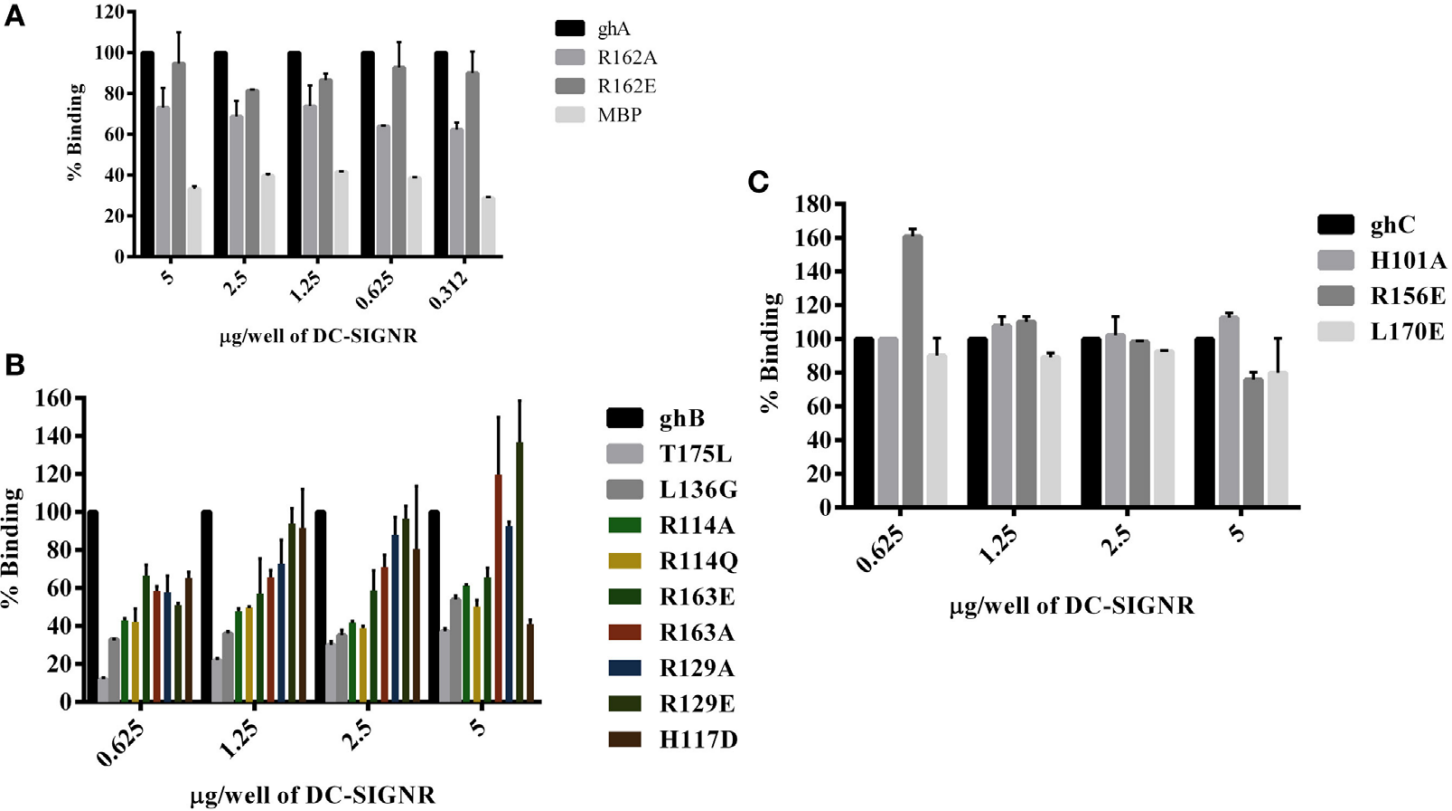
**Binding of globular head substitution mutants to DC-SIGNR**. Different concentrations of DC-SIGNR tetramer were coated on microtiter wells in carbonate buffer overnight at 4°C and then incubated with 2.5 µg/well of recombinant globular head wild type and mutant proteins and probed with anti-MBP monoclonal antibody and rabbit anti-mouse IgG-HRP conjugate. Percent binding was calculated for each mutant using binding of the wild-type globular head module as 100%. **(A)** Binding of ghA, ghA-R162E and ghA-R162A to DC-SIGNR; **(B)** binding of ghB mutants ghB-L136G, ghB-T175L, ghB-R114Q, ghB-R114A, ghB-R163A, ghB-R163E, ghB-R129E, ghB-R129A, and ghB-H117D to DC-SIGNR; **(C)** binding of ghC mutants ghC-R156E, ghC-L170E, and ghC-H101A to DC-SIGNR.

The ability of substitution mutants Arg^A162^Glu and Arg^A162^Ala to bind to DC-SIGN and DC-SIGNR was assessed by ELISA. Both substitution mutants bound DC-SIGN (Figure 4A) and DC-SIGNR (Figure 5A) in a dose-dependent manner. DC-SIGNR was able to interact with Arg^A162^Glu nearly as efficiently as it did with the wild-type ghA, showing a reduction in binding of only 15% at the highest concentration of 5 µg (Figure 5A). Arg^A162^Ala, on the other hand, bound DC-SIGNR with much less affinity, showing a drop of 27% (Figure 5A). Considering DC-SIGN and DC-SIGNR are both highly conserved, Arg^A162^Glu was able to interact with DC-SIGN weakly than it did with DC-SIGNR (Figures 4A and 5A), showing a ~35% reduced binding as opposed to ~5% (seen with DC-SIGNR.) The mutant Arg^A162^Ala bound DC-SIGN in a similar manner as it did to its homologue DC-SIGNR showing a reduced binding of ~25% (Figure 4A).

### ghB Substitution Mutants Bind Differentially to DC-SIGN and DC-SIGNR

Using ELISA, we examined the ability of DC-SIGN to bind to the ghB substitution mutants Arg^B114^Gln, ArgB114Ala, Arg^B163^Glu, Arg^B163^Ala, Arg^B129^Ala, Arg^B129^Glu, His^B117^Asp, Tyr^B175^Leu, and Leu^B136^Gly (29). All the ghB substitution mutants bound DC-SIGN (Figure 4B) and DC-SIGNR (Figure 5B) in a dose-dependent manner. Substituting Arg^B114^ to Gln and Ala resulted in a reduction of ~50% in the case of DC-SIGN (Figure 4B) and DC-SIGNR binding (Figure 5B), suggesting that the Arg residue at this position plays an important role in the C1q–DC-SIGN/DC-SIGNR interaction (Tables 1 and 2).

**Table 1:** **Bindings of globular head modules and its mutants with DC-SIGN (% binding ± SD)**.

ghA	100.00 ± 0.0
R162A	77.71 ± 8.85
R162E	66.41 ± 2.38
ghB	100.0 ± 23.62
R129E	93.91 ± 8.34
R129A	75.05 ± 7.22
R163A	68.76 ± 12.78
R114A	57.37 ± 4.45
R114Q	52.46 ± 1.94
R163E	48.33 ± 8.89
L136G	47.74 ± 9.17
T175L	37.92 ± 7.50
H117D	36.35 ± 10.28
ghC	100.00 ± 0.00
R156E	101.41 ± 0.27
H101A	90.20 ± 2.27
L170E	78.32 ± 1.47

**Table 2:** **Bindings of globular head modules and its mutants with DC-SIGNR (% binding ± SD)**.

ghA	100.00 ± 0.00
R162E	94.78 ± 15.08
R162A	73.17 ± 9.54
ghB	100.00 ± 0.00
R129E	136.70 ± 21.82
R163A	119.68 ± 30.09
R129A	92.55 ± 2.26
R163E	65.69 ± 4.89
R114A	61.44 ± 0.38
L136G	53.99 ± 1.88
R114Q	50.27 ± 3.39
H117D	40.96 ± 2.26
T175L	37.23 ± 1.50
ghC	100.00 ± 0.00
H101A	112.60 ± 2.86
L170E	79.81 ± 20.40
R156E	75.77 ± 4.62

Substituting the ghB mutant Arg^B129^ with Glu and Ala caused a slight reduction of ~20% binding with DC-SIGN (Figure 4B) and up to ~40% with DC-SIGNR (Figure 5B). When Arg^B163^ was replaced with the negatively charged Glu, its affinity for DC-SIGN and DC-SIGNR was reduced by 50 and 35%, respectively (Figures 4B and 5B) (Tables 1 and 2); substitution with Ala resulted in 30% reduction for DC-SIGN (Figure 4B) and increase by 20% for DC-SIGNR (Figure 5B) (Tables 1 and 2). A greater reduction in DC-SIGN and DC-SIGNR binding of ~60% was observed for the ghB mutant His^B117^ substituted for Asp. For the ghB module, Tyr^B175^ substitution to Leu had the most significant effect, showing a dramatic decrease of up to 90% in binding to DC-SIGN as well as DC-SIGNR at a concentration of 0.625 µg; this is not surprising due to its role in stabilizing the gC1q domain.

### Residue Leu^136^ on ghB, Important for IgG Binding, Is Also Involved in DC-SIGN Binding

Using a series of globular head single residue substitution mutants (29), we sought to examine that residues in the ghB chain offered complementary binding sites for DC-SIGN. Since Leu^B136^ and Tyr^B175^ residues are considered important in maintaining the gC1q structure as well as for IgG binding (33), we used Leu^B136^ substituted for Glu and Tyr^B175^ substituted for Leu in direct-binding ELISA. Leu^B136^Gly showed ~50% less binding to DC-SIGN at the highest concentration (Figure 4B), suggesting that DC-SIGN and IgG binding sites on C1q (ghB) are overlapping.

### The Contributions of ghC Substitution Mutants to DC-SIGN Binding

The substitution mutants His^101^Ala, Arg^156^Glu, and Leu^170^Glu bound to DC-SIGN in a dose-dependent manner (Figure 4C). In fact, replacing Leu^170^ with Glu of the ghC chain reduced binding to DC-SIGN with a decrease in ~25% at the highest concentration. The ghC mutants His^101^Ala reduced binding by 10%, suggesting that the contributions of His^101^ and Leu^170^ are comparable in the DC-SIGN–C1q interaction. The ghC substitution mutants also bound to DC-SIGNR in a dose-dependent manner.

The mutants His101Ala appeared to show ~10% better binding to DC-SIGNR with compared to wild type, whereas Leu170Glu and Arg156Glu showed reduced binding by up to 25% at the highest concentration of 5 µg of DC-SIGNR (Figure 5C; Table 2).

### gC1qR and ghB Compete for the Same Binding Site on DC-SIGN

**Figure 6:**
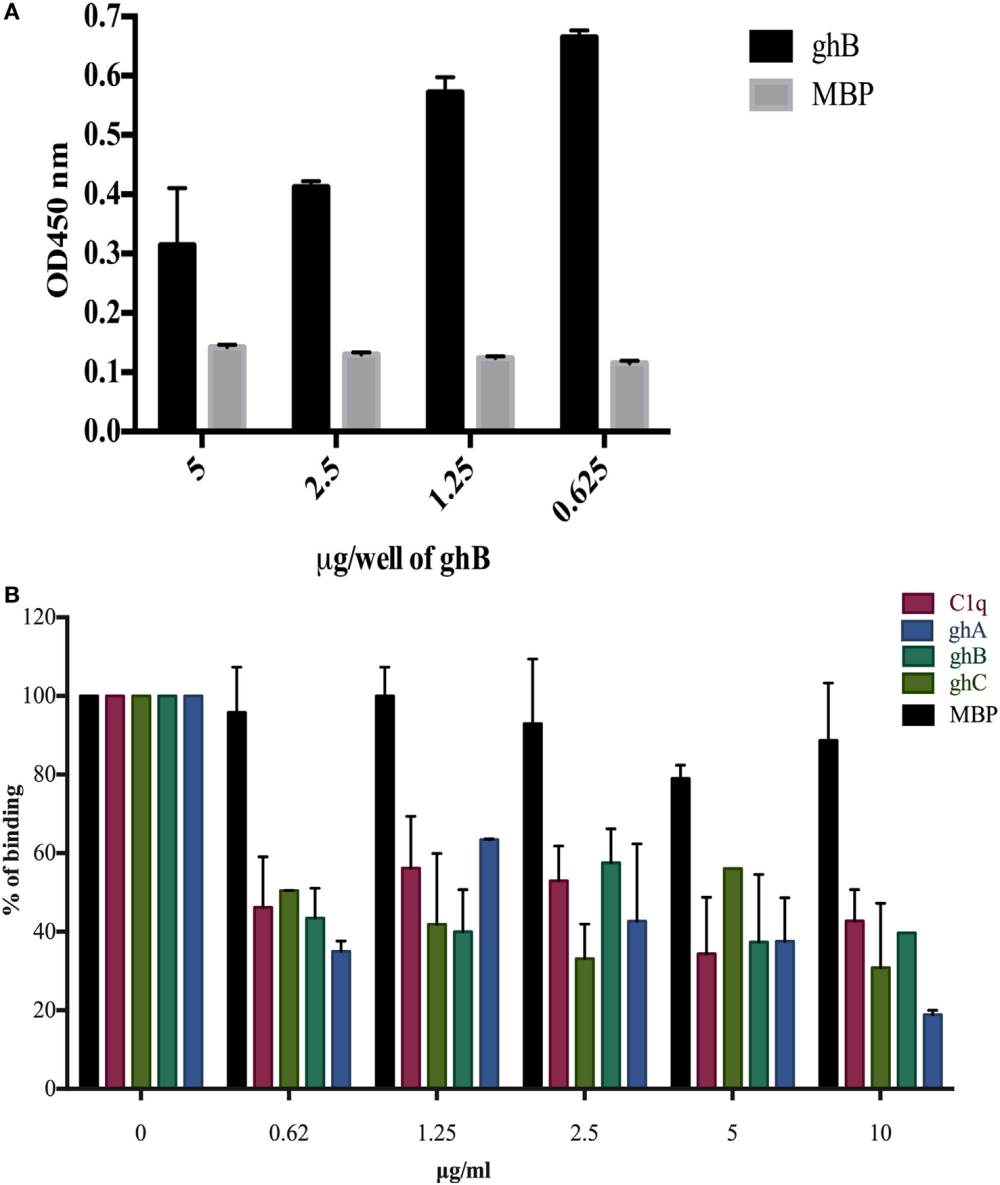
**Competitive inhibition of DC-SIGN: HIV-1 gp120 interaction by globular head modules and gC1qR**. **(A)** ELISA to assess whether gC1qR and ghB directly compete for the same binding site on DC-SIGN: DC-SIGN was coated at 5 µg/well overnight at 4°C. Wells were blocked with 2% BSA in PBS for 2 h at 37°C. gC1qR (5 µg/well) and different concentrations of ghB (5, 2.5, 1.25, 0.625 µg/well) were added in buffer containing 5 mM CaCl_2_. Incubation was carried out at 37°C for 1.5 h and 4°C for 1.5 h. Following repeated washes, bound gC1qR was probed using rabbit anti-gC1qR polyclonal antibodies (1:1,000) and Protein A-HRP (1:1,000). Color was developed using *o*-phenylenediamine dihydrochloride substrate; **(B)** competition between DC-SIGN tetramer and C1q globular head modules to bind solid-phase gp120. Microtiter wells were coated with 250 ng of gp120. Various concentrations of ghA, ghB, ghC, and C1q and constant 2.5 µg/mL of DC-SIGN were incubated at 37°C for 1 h and then at 4°C for 1 h. The binding of DC-SIGN to gp120 in the presence of globular heads or C1q was detected using rabbit anti-DC antibody (1:500), probed with Protein A HRP (1:5,000). DC-SIGN alone binding to gp120 was used as 100%.

In view of the recent report of gC1qR, C1q, and DC-SIGN forming a trimeric complex on immature DCs (15), we examined whether DC-SIGN has complementary and overlapping binding site for C1q and gC1qR. We have recently mapped the gC1qR binding site on ghA, ghB, and ghC (30). Since ghB was found to be the preferential binder of DC-SIGN, ghB modules were tested in a competitive assay. When different concentrations of ghB and a constant concentration of gC1qR were challenged against DC-SIGN, probing with anti-gC1qR polyclonal antibody revealed that with decreasing concentration of ghB, more gC1qR was able to bind to solid-phase DC-SIGN (Figure 6A), thereby implying an overlapping binding site between the proteins. 5 µg of DC-SIGN and 5 µg of gC1qR were able to bind efficiently, showing an OD of 1 (data not shown); this binding appeared to be drastically reduced when 5 µg of gC1qR was allowed to compete with 5 µg of ghB.

### C1q, ghA, ghB, and ghC Inhibit the Binding of DC-SIGN to gp120

C1q, ghA, ghB, and ghC were able to inhibit the binding of DC-SIGN to immobilized gp120 in a dose-dependent manner. The highest concentration of C1q, ghA, ghB, and ghC were able to significantly compete out the binding of DC-SIGN (Figure 6B).

### The ghA, ghB, and ghC Modules Bind to Cell Surface-Expressed DC-SIGN

**Figure 7:**
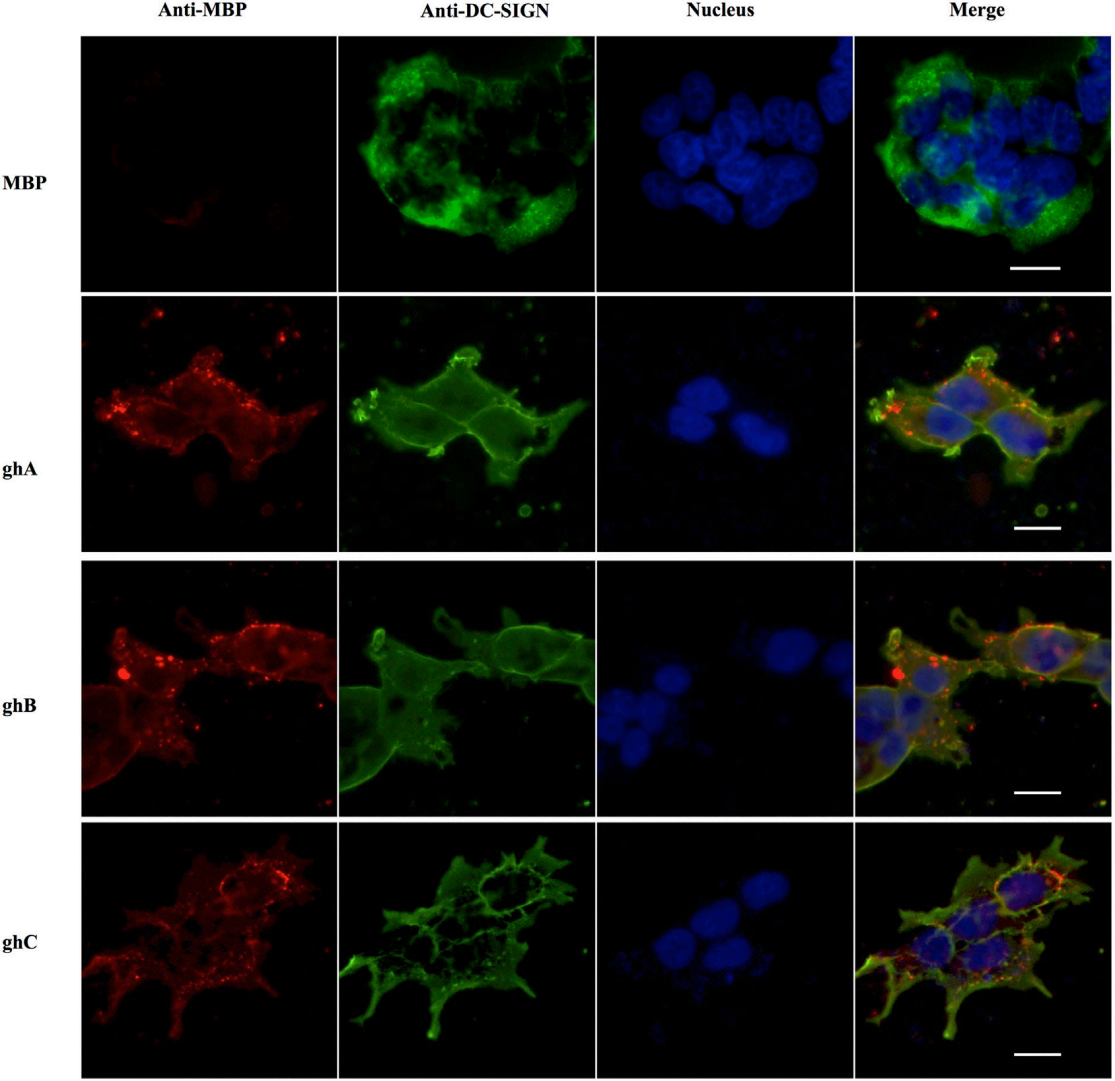
***In vitro* binding of globular heads modules to DC-SIGN expressed on HEK cells**. DC-SIGN-expressing HEK cells (DC-HEK cells) were incubated with recombinant globular head modules (ghA, ghB, ghC) and MBP as control for 30 min at 37°C. DC-HEK cells were fixed with 4% paraformaldehyde, washed and blocked with 5% FCS, and probed with mouse anti MBP antibody to detect the presence of MBP fused globular head modules and rabbit anti DC-SIGN to detect DC-SIGN expressed on the cells. Goat anti-mouse secondary antibody conjugated with Alexa Fluor 568 was used to detect binding of globular heads whereas DC-SIGN expression was visualized using goat anti-rabbit IgG conjugated with Alexa Fluor 488 antibody. Scale bar 20 µm.

The binding of globular head modules to DC-SIGN was also performed using HEK 293 cells expressing DC-SIGN on the cell surface. The surface expression of DC-SIGN on DC-HEK cells was first confirmed with antibodies against DC-SIGN. To confirm the binding of individual globular head modules to DC-SIGN on DC-HEK cells, ghA, ghB, and ghC fused with MBP were added to the DC-HEK cells (Figure 7). Incubation of the globular head modules and probing with anti-MBP monoclonal antibodies showed that each globular head module bound on the surface of DC-HEK cells co-localizing with DC-SIGN expressed on DC-HEK cells unlike MBP (Figure 7).

### C1q Inhibits DC-SIGN-Mediated Transfer of HIV-1 to PBMC in Culture

**Figure 8:**
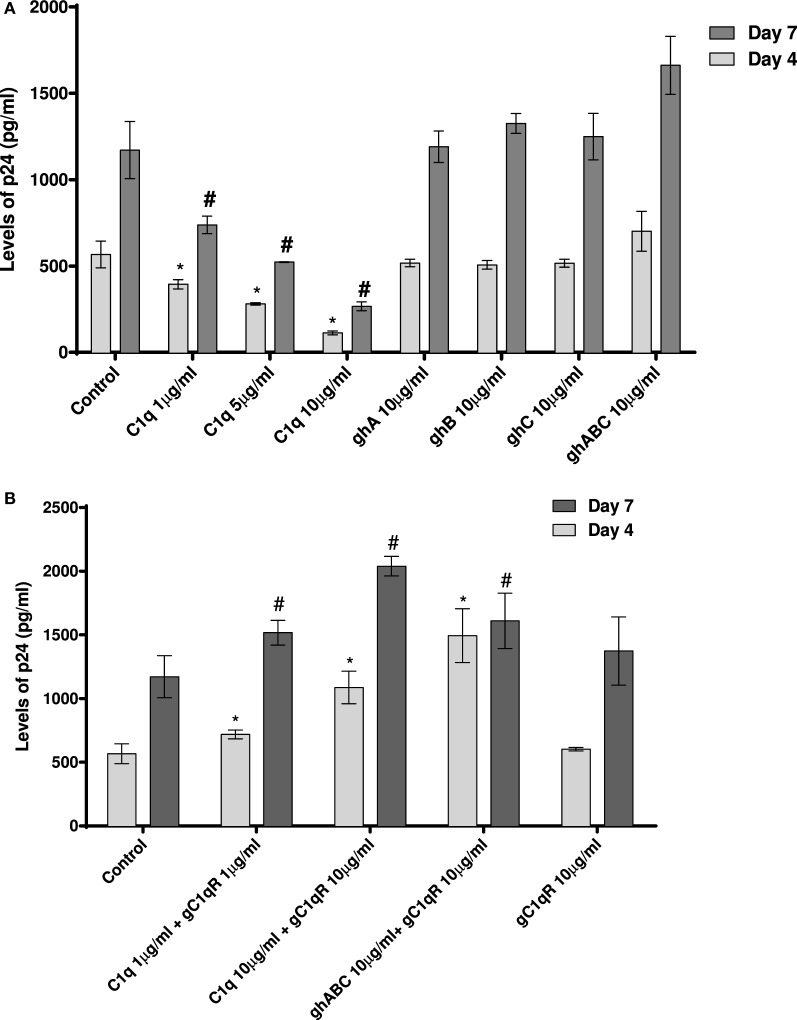
**HIV transfer assay mediated by DC-SIGN**. Cell surface DC-SIGN expressing HEK (DC-HEK) cells were grown in a 12-well plate to form a confluent layer. Different concentrations of proteins were added to the cells and incubated for 2 h for binding. Unbound proteins were removed and cells were challenged with 2.5 ng/mL p24 of HIV-1 (SF-162 strain) for 1 h. Unbound virus was washed off and cells were co-cultured with PHA-activated PBMCs for 24 h. PBMCs were separated from the DC-HEK monolayer and cultured separately for 7 days to determine viral titer of the supernatants collected on days 4 and 7. **(A)** C1q, ghA, ghB, ghC, and ghABC; **(B)** gC1qR in presence of C1q, ghA, ghB, ghC, and ghABC. Data represent mean ± SD. *P* < 0.05 is considered significant. * and # indicate statistical significance in comparison to untreated controls of days 4 and 7, respectively.

Since CD4^+^ T cells and macrophages are the main cells targeted by HIV-1, we looked at the potential of C1q, ghA, ghB, ghC, and gC1qR to modulate DC-SING-mediated transfer of HIV-1 to activated PMBCs. As shown in Figure 8A, C1q considerably inhibited viral transfer to PBMCs in a dose-dependent manner on days 4 and 7. The globular head modules, ghA, ghB, and ghC, surprisingly did not interfere with HIV-1 transfer, neither individually nor collectively, when compared to untreated or MBP-treated control, suggesting that the collagen region of C1q and/or its multivalency of the gC1q domains are likely requirement for enforcing inhibitory properties. Addition of MBP in the control wells did not significantly affect the p24 levels in comparison with untreated controls (data not shown). Furthermore, during the assay period, cellular viability was not affected by any of the protein treatments, suggesting that differences in the infectivity were not due to cell death (data not shown).

We further examined the involvement of gC1qR with DC-SIGN in HIV-1 infection transmission (Figure 8B). gC1qR has previously been shown to inhibit CD4–gp120 interaction in HIV-1 isolates. It has also been recognized as a receptor on CD4^+^ T cells that gp41 engages with in order to cause death of bystander CD4^+^ T cells. We wanted to determine the role of gC1qR in DC-SIGN-mediated infection with or without C1q. Figure 8B shows that gC1qR alone did not mediate viral transfer through DC-SIGN but significantly promoted viral transfer in the presence of C1q as well as the three globular head modules together for up to 7 days. This suggests that the tripartite interaction between C1q, gC1qR, and DC-SIGN enhances DC-SIGN-mediated viral transfer. Thus, association of these proteins on DCs may actually promote HIV-1 infection. In addition, involvement of gC1qR in the tripartite complex is likely to negate the protective effect of C1q.

## Discussion

The role of complement in HIV-1 pathogenesis is well-documented. Complement-opsonized HIV-1 causes enhanced viral infection of CD4^+^ T cell lines (34), PBMCs, monocytes, and macrophages (35). DC-SIGN on the surface of immature DCs is involved in the capture of C3 opsonized R5 and X4 tropic HIV-1 and enhanced transmission to T cells (36). C1q can bind to gp41 directly in an antibody-independent manner and activate the classical pathway (16); however, this leads to an enhanced infection of complement receptor-bearing cells (37). To escape complement-mediated destruction, HIV-1 uses follicular DCs as a viral reservoir (38), following its internalization *via* CR2, and remains within its protective recycling endosome. Following emergence from the endosome to the cell surface, HIV-1 infects follicular T cells through CD4. HIV-1 opsonized with C3b interacts with CR1 on erythrocytes with factor I dissociating erythrocytes from this complex and converting C3b to iC3b and C3d. C3d opsonized HIV-1 is then able to bind to CR2 on B cells (39). In this study, we have examined complement-independent interaction of C1q with HIV-1 *via* DC-SIGN.

C1q is a charge pattern recognition protein that binds to a variety of ligands *via* its gC1q domain (19). Following its ability to interact with DC-SIGNR (32), Hosszu et al. recently have shown that C1q also recognizes a peptide derived from its homolog DC-SIGN (15). In the current study, we made use of the availability of recombinant individual globular head modules of C1q (ghA, ghB, and ghC) and its substitution mutants to establish that C1q binds DC-SIGN (and DC-SIGNR) *via* its gC1q domain.

Structure-function studies have demonstrated that the CRD region of DC-SIGN is the specific site for ligand binding and only funcions in the presence of the neck region within the ECD (12). We performed a series of binding experiments involving both the tetrameric forms of DC-SIGN and DC-SIGNR (comprising of the ECD and CRD region) as well as the monomeric forms, which only consist of the CRD region. We asked the question whether DC-SIGN and DC-SIGNR binding sites for C1q (and its gC1q domain) lies within their CRD region, or the α-helical neck region also plays an important role in these interactions. Both proteins have an increased affinity for glycoproteins containing high mannose oligosaccharides, such as mannan (40), gp120 (10), and ICAM-3 (41), *via* the CRD region. Here, individual globular head modules bound better to the tetrameric forms of DC-SIGN and DC-SIGNR as opposed to just the CRD region alone, indicating that the neck region, and hence, multimerization is needed to facilitate C1q binding. The neck region of DC-SIGN and DC-SIGNR interestingly differ most in their α helical structures (8) as the 23 amino acid repeats only show the first half of each repeat presenting a pattern of hydrophobic residues spaced at intervals, a feature that is abundant in most dimeric and trimeric coiled-coils (42).

We also found that the ghB module bound better to DC-SIGN and DC-SIGNR. Previous studies have identified ghB as a key module of the gC1q domain in binding to IgG, PTX3, and CRP (43, 44). Interestingly, C1q and ghB bound DC-SIGNR better than DC-SIGN, despite 77% sequence similarity. We also expressed and purified globular head mutants (29) where single amino acid residues were substituted in order to localize key residues involved in C1q interaction with its various ligands. These mutants were designed based on the crystal structure and are the residues known to be important in binding to various C1q ligands (43). It appears that IgG and DC-SIGN binding sites on ghB are overlapping and shared, including Lys^136^ and Tyr^175^ on ghB, which have been previously shown to be important for binding to IgG (29) and for gC1q assembly (45). We also examined the roles of Arg^114^, Arg^129^, and Arg^163^ of the ghB module since arginine residues have previously been shown to be important for the C1q–IgG interaction (46). Moreover, Hosszu et al. have reported that C1q binds DC-SIGN *via* its IgG binding site (15). Our results highlighted the significance of Arg^114^ in C1q interaction with DC-SIGN and DC-SIGNR (Figures 4B and 5B). Substituting Arg^114^ with the polar residue Glutamine and hydrophobic residue Ala led to ~80% reduction, highlighting a very important role for Arginine^114^ of B chain in C1q–DC-SIGN interaction. In addition, Tyr^175^ appears critical for C1q interaction with DC-SIGN and DC-SIGNR (Figures 4B and 5B); the binding analysis revealed a dramatic reduction (82% for DC-SIGN and 90% for DC-SIGNR following substitution of Tyr with Leu). This is not the first time Tyr^175^ has been shown to be a critical residue in gC1q binding (33). Gadjeva et al. have shown that this residue mainly constitutes C1q binding to IgM. Overall, our binding studies suggest that Tyr^175^ and Arg^114^ of ghB are critical for the C1q–DC-SIGN and C1q–DC-SIGNR interaction.

The known dual roles of DC-SIGN as a facilitator of adaptive immune response as well as promoter of HIV-1 infection prompted us to examine if innate immune soluble factors such as C1q and gC1qR can potentially modulate viral transmission *via* DC-SIGN (47), similar to reports involving CD4^+^ T cells (48) and a lectin drug GRFT (*Griffithsia)* isolated from the red algae (49). We also included DC-SIGNR (DC-SIGN-related), a homolog of DC-SIGN, in our study. DC-SIGN-R is expressed on endothelium including liver sinusoidal (50), lymph node sinuses, and placental capillary (8). DC-SIGNR can bind ICAM-3 as well as gp120 to facilitate HIV-1 viral infection (50). As a receptor for bacterial dextrans (51) and capsular pneumococcal polysaccharide of *Streptococcus pneumoniae*, DC-SIGN-R can cause proteolysis of C3 (32). DC-SIGN-R is shown to be highly expressed by spleen marginal zone macrophages (MZM) and lymph node macrophages (52). SIGN-R1 (mouse homologue) in MZM interacts with C1q in the spleen and enhances apoptotic cell clearance *via* activation of the classical pathway (53).

The transmembrane envelope gp41 protein of HIV-1 is known to interact with C1q (54) through its A chain (19), leading to complement activation but no viral lysis (55). Instead, the virus is transmitted to complement receptor bearing cells such as macrophages and CD4^+^ T cells allowing infection to take place (37, 54, 56, 57). HIV-1-infected CD4^+^ T cells can activate the classical pathway *via* shedding of gp120, leading to unmasking of the gp41 epitope 601–613 available for interaction with C1q (23). C1q is also involved in a range of processes independent to its complement functions (58, 59), including DC differentiation (60). C1q, along with its globular head receptor gC1qR and DC-SIGN, can co-localize on the surface of blood precursor DCs to promote DC differentiation (15). gC1qR, a multifunctional pathogen recognition receptor (61, 62), can also interact with gp41 of HIV-1 (17) on uninfected CD4^+^ T cells and upregulate NK cell ligand NKP44-L, rendering healthy CD4^+^ T cells susceptible to NK cell lysis. Since DC-SIGN is a receptor for HIV-1 through its binding to gp120, it is interesting that it co-localizes with C1q and gC1qR, the two proteins, also known for HIV-1 binding and transmission of the viral infection. Such association forming a trimolecular unit on the target cell surface may create a vehicle that promotes pathogen entry and immunosuppression (15).

**Figure 9:**
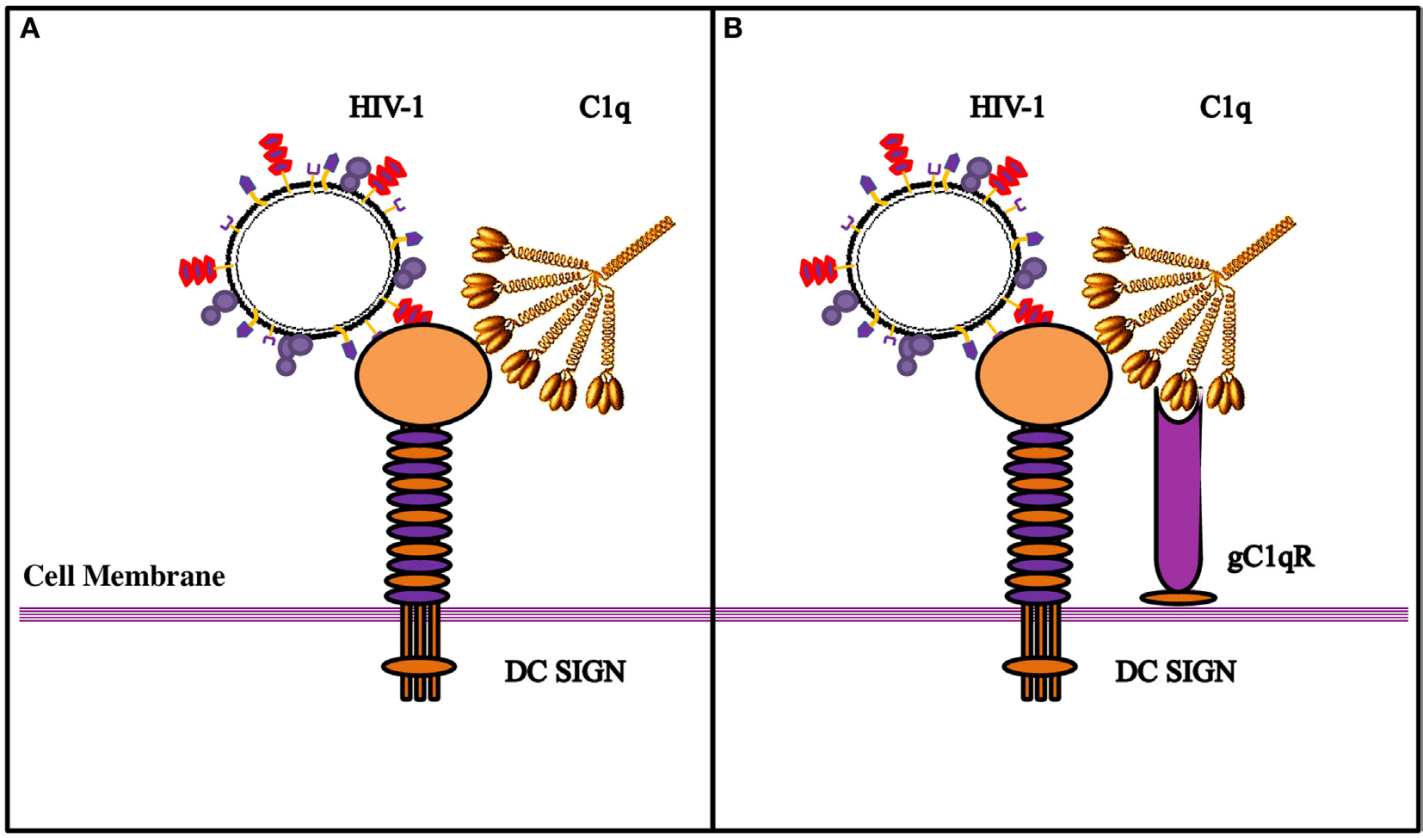
**Diagrammatic model explaining the possible implications of the tripartite molecular interplay between DC-SIGN, C1q, and gC1qR**. **(A)** By virtue of its ability to bind to DC-SIGN on the cell surface, C1q is likely to inhibit interaction between DC-SIGN and HIV-1 gp120, resulting in the inhibition of viral transfer. **(B)** On the DC/Monocyte surface, a trimolecular receptor complex is formed between gC1qR, C1q, and DC-SIGN. Although each of these molecules can bind the HIV-1 virus independently, we postulate that it is the binding of the HIV-1 gp-41 to both gC1qR and C1q that initiates the membrane fusion before the final binding of gp120 to DC-SIGN and/or CD4, eventually allowing the internalization of the virus. It is possible that HIV-1 interaction with DC/monocytes causes recruitment of gC1qR to the cell surface, or its secretion, which in turn, can bind to C1q globular heads, thereby neutralizing the protection offered by C1q.

We wanted to examine if C1q–DC-SIGN interaction modulated HIV-1 transfer. We found that full length C1q but not its individual globular heads, suppressed DC-SIGN-mediated HIV-1 transfer to activated PBMCs. Curiously, addition of gC1qR negated the protective effects of C1q by enhancing DC-SIGN-mediated viral transfer. gC1qR, as an inhibitor of HIV-1 infection, can block the interaction between CD4 and gp120 and prevent viral entry (24). Since DC-SIGN binds to gp120 and gC1qR to gp41, both promoting infection, we can consider that even if gC1qR does interfere with the DC-SIGN-gp120 interaction, its active binding site for gp41 is still available to facilitate infection. The increased viral transmission of gC1qR seen when in association with C1q suggests that C1q bound to gC1qR can enhance its function. It is possible that C1q plays a protective role by blocking access of gp120 to DC-SIGN (Figure 9). This can happen if C1q shares binding sites on DC-SIGN. The globular heads, individually or in combination, did not appear to inhibit virus transmission unlike full length C1q, suggesting that the collagen domain of C1q and/or probably oligomeric form of C1q is required for the observed inhibitory effect.

In summary, we found that gC1qR can alter C1q–DC-SIGN interaction in a way that it promotes viral transfer, thus neutralizing the protective effect of C1q. The tripartite complex involving DC-SIGN–gC1qR-C1q probably leads to an increase in the distance between DC-SIGN and C1q that permits DC-SIGN interaction with gp120; this allows DC-SIGN and gC1qR to bind to the virus with enhanced affinity (Figure 9).

## Author Contributions

LP, HP, BP, and AK carried out crucial experiments; MA-M, LK, BG, and DM provided important reagents; TM supervised few experiments and helped with manuscript preparation; UK led the research, conceived the experiments, and drafted the manuscript.

## Conflict of Interest Statement

The authors declare that the research was conducted in the absence of any commercial or financial relationships that could be construed as a potential conflict of interest.
